# Combined subsoiling and ridge–furrow rainfall harvesting during the summer fallow season improves wheat yield, water and nutrient use efficiency, and quality and reduces soil nitrate-N residue in the dryland summer fallow–winter wheat rotation

**DOI:** 10.3389/fpls.2024.1401287

**Published:** 2024-06-06

**Authors:** Jinzhi Wu, Rongrong Wang, Wenxin Zhao, Kainan Zhao, Shanwei Wu, Jun Zhang, Hezheng Wang, Guozhan Fu, Ming Huang, Youjun Li

**Affiliations:** College of Agriculture, Henan University of Science and Technology, Luoyang, China

**Keywords:** dryland, wheat, tillage practice during summer fallow season, grain yield, productivity, nitrate-N residue

## Abstract

Both subsoiling tillage (ST) and ridge and furrow rainfall harvesting (RF) are widely implemented and play an important role in boosting wheat productivity. However, information about the effects of ST coupled with RF during the summer fallow season on wheat productivity and environmental issues remains limited. This study aims to explore the effects of ST coupled with RF on water harvesting, wheat productivity–yield traits, water and nutrient use efficiency and quality, and soil nitrate-N residue in dryland winter wheat–summer fallow rotation at the intersection of southern Loess Plateau and western Huang–Huai–Hai Plain in China in 2018–2022. Three tillage practices—deep plowing with straw turnover (PTST), subsoiling with straw mulching (STSM), and STSM coupled with RF (SRFSM)—are conducted during the summer fallow season. The results indicated that tillage practices during the summer fallow season significantly impacted wheat productivity and soil nitrate-N residue. Compared to PTST, STSM significantly enhanced rainfall fallow efficiency and water use efficiency by 7.0% and 14.2%, respectively, as well as N, P, and K uptake efficiency by 16.9%, 16.2%, and 15.3%, and thus increased grain yield by 14.3% and improved most parameters of protein components and processing quality, albeit with an increase in nitrate-N residue in the 0- to 300-cm soil depth by 12.5%. SRFSM, in turn, led to a further increase in water storage at sowing, resulting in an increase of water use efficiency by 6.8%, as well as N, P, and K uptake efficiency and K internal efficiency by 11.8%, 10.4%, 8.8%, and 4.7%, thereby significantly promoting grain yield by 10.2%, and improving the contents of all the protein components and enhancing the processing quality in grain, and simultaneously reducing the nitrate-N residue in the 0- to 300-cm soil layer by 16.1%, compared to STSM. In essence, this study posits that employing subsoiling coupled with ridge–furrow rainfall harvesting (SRFSM) during the summer fallow season is a promising strategy for enhancing wheat yield, efficiency, and quality, and simultaneously reducing soil nitrate-N residue within the dryland summer fallow–winter wheat rotation system.

## 1 Introduction

Wheat (*Triticum aestivum L.*), which accounts for more than 20% of the world’s arable land, contributes to over 45% of the global calorie supply and over 20% of the global protein supply, and feeds approximately 30% of the world population (FAO, 2016), plays a crucial role in food security and people’s dietary structure optimization. However, approximately 75% of all the wheat is produced from the dryland including arid, semi-arid, and semi-humid drought-prone areas (Zhang et al., 2019). In these areas, water shortage, infertile soil, and less-advanced cropping technique are the critical limiting factors for sustaining the wheat production (Hartmann et al., 2015; Sun et al., 2018; Wang and Li, 2019; Huang et al., 2021), which not only result in a low and unsustainable yield, efficiency, and quality (Huang et al., 2021), but also lead to environmental issues such as nitrate-N leaching and the emission of nitrous oxide and ammonia due to the high amount of nitrate-N residue in wheat fields (Zhou and Buterbach-Bahl, 2014; Lu et al., 2019; Zhao et al., 2023a, Zhao et al., 2023b). The summer fallow–winter wheat (namely, F–W) is one of the most popular wheat cropping systems in the dryland area of China, where the winter wheat is planted in the beginning of late September to October and harvested in late May to early July in the next year, followed by summer, which is approximately 3–4 months before the next sowing of winter wheat (Wang and Li, 2019). In this system, the annual rainfall is 400–800 mm, with 60%–70% occurring during the summer fallow season, while the rainfall during the wheat growth period is usually approximately 200 mm, which cannot meet the water needs of wheat growth (Shi et al., 2021; You et al., 2022). This situation has led to a lower, unstable grain yield, lower resource efficiency in wheat (Cao et al., 2017; Wang and Li, 2019), and overaccumulation of nitrate-N in soil (Dai et al., 2015; Huang et al., 2017). Therefore, sustainable agricultural practices are required to increase wheat yield, efficiency, and quality (Adel et al., 2019) and reduce the soil nitrate-N residue in the F–W system (Wang and Li, 2019).

Subsoiling tillage [ST, usually combined with straw mulching (STSM)] plays important roles in agricultural production, especially in dryland regions, as it is able to increase rainfall harvest, crop yield, and efficiency (Cai et al., 2014; Arnhold et al., 2023) and address environmental issues (Zhao et al., 2023b). Previous studies have primarily demonstrated that ST can influence crop growth and development by reducing soil bulk density (Ahmad et al., 2009; Lampurlanés et al., 2016; Sun et al., 2018), increasing soil porosity (Xue et al., 2018), enhancing water infiltration (Liang et al., 2019; Qiang et al., 2022), increasing soil water usage (Wang et al., 2022; Yang et al., 2022), optimizing soil physical properties and fertility (He et al., 2019; Wang et al., 2020; Yang et al., 2022), improving root characteristics (Izumi et al., 2009; He et al., 2019; Koch et al., 2021; Arnhold et al., 2023), enhancing tiller density (Lv et al., 2019), delaying senescence (He et al., 2020), promoting plant photosynthesis characteristics (Sang et al., 2016; He et al., 2019), and facilitating dry matter accumulation and remobilization (Zhang et al., 2023). In the Loess Plateau of China, studies of two 2-year experiments showed that ST during the summer fallow season improved soil nutrient characteristics, wheat growth, and N uptake characteristics, thus not only increasing wheat yield and water use efficiency (WUE), but also optimizing the quality of albumin, gliadin, glutenin, total protein, and sedimentation values and wet gluten contents (Sun et al., 2013; Zhao et al., 2017). In the Huang–Huai–Hai Plain in China, a field experiment also showed that ST combined with strip rotary promoted the absorption of nitrate-N by wheat, enhancing N accumulation from jointing to maturity (particularly from anthesis to maturity), thus increasing N accumulation in grains and grain yield (Wang et al., 2015). In the study area, our previous studies found that STSM during the summer fallow season increased the soil water storage during sowing (Wu et al., 2021), enhanced shoot and grain N accumulation, improved grain yield and water and N use efficiency in wheat, and reduced the nitrate-N residue at harvest (Huang et al., 2021).

In addition to ST, the ridge and furrow rainfall harvesting technique (RF) is widely utilized in dryland regions to boost crop yield (Zhang et al., 2016; Ali et al., 2017; Zhang et al., 2018; Wu et al., 2023). In the RF system, the arrangement of alternating parallel ridges and furrows helps to channel rainfall water into the furrows, facilitating easy infiltration into deeper soil layers and aiding in rainwater and runoff collection. Moreover, RF has demonstrated the effective regulation of soil water content, temperature, and nutrients (Li et al., 2012; Li et al., 2013; He et al., 2016a, c; Zhang et al., 2021); enhanced soil enzymatic activity and microbial abundance (Zhang et al., 2022); increased root biomass, root length density, and root surface density in the root concentrated layer (Hu et al., 2020); elevated leaf area index, leaf chlorophyll content, and net photosynthetic rate (Gu et al., 2021); promoted plant growth, nutrient absorption, and dry matter accumulation (Ali et al., 2017; Zhang et al., 2022); and ultimately markedly improved grain yield and WUE in wheat (Gan et al., 2013; Chen et al., 2022; Zhang et al., 2022). Previous studies have confirmed that employing the FS technique can markedly reduce the soil nitrate-N nitrate content in the growth period for spring maize (Zhang et al., 2021), compared to the flat planting system. Another study showed that an RF soil surface—formed by previous wheat production—during the summer fallow season increased rainfall harvesting and forced nitrate-N leaching into the deep soil layer (He et al., 2016a, He et al., 2016c). In the study area, we also found that the RF soil surface formed by the previous wheat production helps to increase rainfall harvesting, nutrient efficiency, and grain yield of wheat (Yang et al., 2021). In addition, the combined tillage selection and ridge–furrow technique can facilitate root proliferation and increase nitrogen accumulation, translocation, and grain yield of maize in the dryland area (Zhang et al., 2023).

To the best of our knowledge, few studies have focused on the combination of ST and RF during the summer fallow season on crop productivity and soil nitrate-N residue. Therefore, we proposed a novel technique that couples ST and RF with straw mulching (namely, SRFSM) during the summer fallow season and employs deep plowing with straw turnover (PTST), subsoiling with straw mulching (STSM), and SRFSM in a 4-year field experiment in a semi-humid, drought-prone region of China. This research aims to (1) investigate the effects of ST and SRFSM on grain yield, water and fertilizer use efficiency, and grain quality; (2) assess the effects of ST and SRFSM on nitrate-N residues; and (3) identify an optimized adaptive tillage technique during the summer fallow season based on the synergistic effect of crop yield, efficiency, quality, and soil nitrate-N in drylands.

## 2 Materials and methods

### 2.1 Study site description

**Figure 1:**
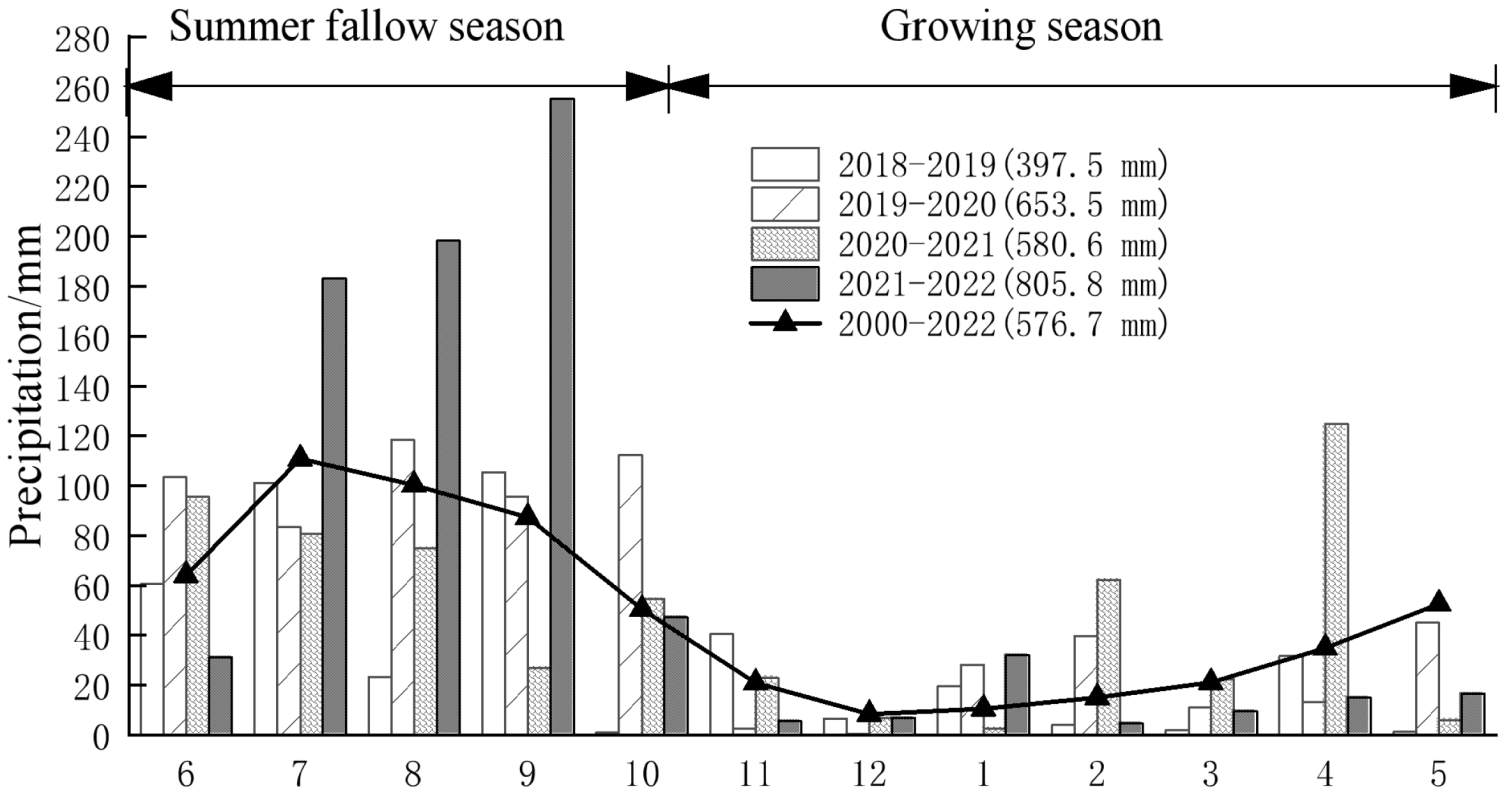
Monthly precipitation in the experimental site from June 2018 to June 2022.

From June 2018 to June 2022, a 4-year field experiment was conducted at Meiyao village (111°71′ E, 34°47′ N) in Xiaojie town of Luoning county, Luoyang, Henan province, which is a typical dryland summer fallow–winter wheat production area at the intersection of the southern Loess Plateau and the western Huang–Huai–Hai Plain of China. The average local annual air temperature is 13.7°C, the mean annual frost-free period is 216 days, the average annual amount of sunshine is 2,218 h, and the average annual precipitation is 577 mm. Approximately 70% of the annual precipitation occurs between June and September, which is slightly misaligned with the winter wheat growing season. The summer fallow–winter wheat is one of the main cropping systems in the study area, which involves planting winter wheat in early to mid-October and harvesting it in early June the following year. The annual precipitation levels were 397.5 mm, 653.2 mm, 580.6 mm, and 805.8 mm, and categorized as dry, normal, normal, and wet for the years 2018–2019, 2019–2020, 2020–2021, and 2021–2022, respectively. During these periods, 73.1%, 68.6%, 52.5%, and 88.8% of the total precipitation were received in the summer fallow season (**Figure 1**). The soils at the experimental site were formed from cinnamon parent material and classified as calcareous Eum-Orthic Anthrosol according to the Chinese soil taxonomy. A field with uniform fertility was chosen, and consistent soil management practices were implemented to maintain soil fertility balance since October 2016. Upon commencing the experiment in 2018, the basic properties of the 0- to 20-cm soil layer were as follows: pH 8.2, organic matter content 12.5 g·kg^−1^, total nitrogen content 0.8 g kg^−1^, alkali–hydrolyzable nitrogen content 15.7 g·kg^−1^, available phosphorus content 23.2 mg kg^−1^, and available potassium content 197.33 mg kg^−1^.

### 2.2 Experimental design and field management

**Figure 2:**
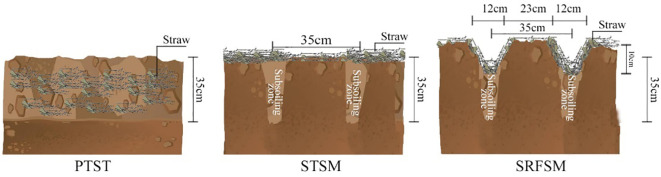
Schematic diagram of PTST, STSM, and SRFSM during the summer fallow season.

Because tillage practice is difficult to do in a small plot, a large-area design with three tillage practices was applied during the summer fallow season according to Zhao et al. (2018). Three tillage practices included conventional practice of plowing tillage with straw turnover (PTST), subsoiling with straw mulching (STSM), and STSM coupled with RF (SRFSM), which are shown in **Figure 2**. For PTST, following local farming practices, the previous wheat crop was harvested with 15–20 cm of stubble, and all the straw was evenly spread on the ground. Subsequently, deep plowing to a depth of 35 ± 3 cm was carried out after receiving adequate rainfall around late July or early August, incorporating the straw into the soil through plowing tillage. In the case of STSM, similar to PTST, the previous wheat crop was harvested with 15–20 cm of stubble, and the straw was uniformly distributed on the ground. Subsoiling to a depth of 35 ± 3 cm, with a 35-cm interval following conventional practices, was performed approximately 2 weeks after the previous wheat harvest. As for SRFSM, the management practices regarding subsoiling and straw were identical to those of STSM. Additionally, a furrow was created alongside the subsoiled zone, formed during the subsoiling and ridging process, while a ridge was established parallel to the non-subsoiled area. Subsequently, a ridge with a width of 23 ± 2 cm and a height of 10 ± 2 cm, along with a furrow measuring 12 ± 2 cm in width and 10 ± 2 cm in depth, was constructed in the field. Since July 2018, all the treatments have been conducted in the fixed area. The area of each treatment was 420 m^2^ (60 m × 7 m). At the four-leaf stage of wheat in each growing season, three 35 m^2^ (7 m × 5 m) representative sampling areas were selected for each treatment; thus, there were three replicates in each treatment.

In 2018–2019 and 2019–2020, wheat was drill-planted in furrows spaced 14 cm apart using a ridge and furrow seeder (2BMQF-6/12A, Luoyang Xinle Machinery Co., Ltd., Luoyang, China). Following sowing, ridges and furrows were formed in the field. The ridge had a width of 20 cm and a height of 10 cm, while the furrow was 14 cm wide. This setup resulted in a 20-cm spacing for wheat in the wide rows and a 14-cm spacing in the narrow rows, with an average row spacing of 17 cm. In 2021–2022 and 2022–2023, wheat was drill-planted with an equal row spacing of 20 cm using a conventional flat seeder (2BJM-6, Henan Haofeng Machinery Co., Ltd., Xuchang, China). In all treatments, 750 kg hm^−2^ of compound fertilizer (N:P_2_O_5_:K_2_O = 25:12:8) was evenly broadcast 3 days before sowing and incorporated into the top 0- to 15-cm soil layer as a basal application using a rotary fertilizer seeder. In addition to the changes made to the cultivar and sowing method, wheat cultivation managements were consistent across all treatments except for tillage practices during the summer fallow season. Details regarding the dates of tillage, sowing, and harvest, as well as information on cultivars and seeding amounts, are provided in **Table 1**. No extra irrigation was supplied in addition to natural precipitation. Weeds, pests, and diseases were controlled with herbicides and pesticides according to the practices employed by local farmers.

**Table 1:** The date of tillage, sowing, and harvest, and wheat cultivars and seeding amount in 2018–2022.

Year	The date of tillage		Cultivars	Seeding amount (kg ha^−1^)	Sowing date (d-m)	Sowing method	Harvest date (d-m)
	PTST	STSM, SRFSM					
2018–2019	29/7	17/6	ZM175	187.5	9/10	Furrow seeding	7/6
2019–2020	3/8	15/6	LH22	187.5	13/10		7/6
2020–2021	31/7	23/6	LH22	225.0	15/10	Strip seeding	5/6
2021–2022	10/8	19/6	LH22	225.0	29/10		6/6

### 2.3 Measurements and methods

#### 2.3.1 Soil water

The soil gravimetric water content (SWC) was determined before the experiment was initiated and periodically at sowing and maturity in 2018–2022. Three random core samples were collected using a handheld soil ferric auger (inner diameter = 4.0 cm) from each sampling plot at a soil depth of 0–200 cm with intervals of 20 cm. The soil samples from the same layer in the same plot were merged, and approximately 300 g of thoroughly mixed soil was sealed immediately in a marked plastic bag for subsequent analysis. The soil water content was determined gravimetrically by drying in an oven at 105°C for 24 h.

Soil water storage (SWS, mm) was calculated using the following equation (Zhao et al., 2023a, b):


$$\text{SWS}=\sum_{\text{i}=20}^{\text{n}}{\text{ D}}_{\text{i}}\times {\text{H}}_{\text{i}}\times {\text{W}}_{\text{i}}\times 10÷100$$

where D_i_ is the soil bulk density (g cm^−3^); H_i_ is the soil thickness of the i layer (cm); W_i_ is the soil water content on a gravimetric basis (%); and n is the number of soil layers; i = 20, 40, 60,…, 200. The soil bulk density of 1.32, 1.34, and 1.38 g cm^−3^ was used in the 0- to 20-, >20-to 40-, and >40-cm-deep soil layer, respectively, according to the average value of the local field.

Water harvesting (WH, mm) during the summer fallow season was calculated as (He et al., 2016c):


$$\text{WH}={\text{SWS}}_{\text{s}}-{\text{SWS}}_{\text{p}}$$

where SWS_p_ and SWS_s_ are the SWS in the 0- to 200-cm soil layer at maturity of the previous wheat and sowing of the present wheat, respectively.

Evapotranspiration (ET, mm) over the whole winter wheat growing season was calculated as (He et al., 2016c; Zhao et al., 2023b):


$$\text{ET}={\text{SWS}}_{\text{s}}+\text{P}+\text{I}+\text{U}-\text{R}-\text{F}-{\text{SWS}}_{\text{m}}$$

where SWS_s_ and SWS_m_ are the SWS in the 0- to 200-cm soil layer during sowing and at maturity, respectively. P (mm) is the precipitation during wheat growth period; I (mm) is the irrigation amount; U is the upward flow into the root zone; R is the surface runoff; and F is the downward drainage out of the root zone. In our experiment, because the plots are even and no irrigation is applied, the groundwater is below more than 10 m of soil layer. Therefore, I, U, R, and F are zero in this study.

#### 2.3.2 Soil nitrate-N

Three random core samples were collected from each sampling plot at a soil depth of 0–300 cm with intervals of 20 cm and at 0–60 cm with intervals of 30 cm at maturity in 2020–2021 and 2021–2022. The sample procedures were the same as in Section 2.3.1. A subsample of 5.0 g was extracted with 50 mL of 1 mol L^−1^ KCl by shaking for 1 h (Dai et al., 2015), and the nitrate-N concentrations in the filtrate were determined immediately with a high-resolution digital colorimeter Auto Analyzer 3 (AA3, SEAL Company, Germany).

The soil nitrate-N residue (NR, kg N ha^–1^) in the 0- to 300-cm soil layer was calculated as follows (Dai et al., 2015):


$$\text{NR}=\sum_{\text{i}=20\text{ or }30}^{\text{n}}{\text{ D}}_{\text{i}}\times {\text{H}}_{\text{i}}\times {\text{C}}_{\text{i}}\times 10÷100$$

where D_i_ is the soil bulk density (g cm^−3^), H_i_ is the soil layer thickness (cm), and C_i_ is the soil nitrate concentration (mg kg^−1^), i.e., i = 20, 40, 60, 90, 120, 150, 180, 210, 240, 270, and 300; 10 and 100 are the conversion coefficients.

#### 2.3.3 Grain yield, yield components, and harvest index

At maturity, three 1 m × 1 m sampling areas were selected randomly in each sample plot and the plants were harvested manually to determine the grain yield. After air drying, the sampled plants were threshed and the grain was weighed. In addition, three 0.5-m-long portions of the winter wheat samples were cut from three different rows in each sampling plot, and the grains per spike and 1,000-grain weight were determined. After cutting off the root, samples were separated into three components (stem + sheath + leaf, rachis + glume, and grain) at maturity. Subsamples of approximately 50 g of air-dried grain and 30 g of air-dried straw or glume were also oven-dried at 60°C to determine the water content. For each sampling plot, the grain yield was expressed at a moisture content of 13.0%, and the biomass yield was expressed on a dry weight basis and calculated according to the air-dried weight and its water content. HI was determined as the ratio of the grain yield relative to the biomass yield.

#### 2.3.4 Plant N, P, and K uptake

In 2020–2021 and 2021–2022, the oven-dried samples of grain, straw, and glume were ground with a ball miller (MM400, RETSCH, Germany) and then digested with H_2_SO_4_–H_2_O_2_. The N and P concentrations in the digest solution were determined using an AutoAnalyzer 3 (AA3, Seal Company, Germany) and K concentration was measured using a flame spectrophotometer (Flame Photometer 410, Sherwood Company, England). The nutrient (N, P, and K) uptake levels in each organ were calculated as the dry weight (kg ha^−1^) multiplied by the corresponding nutrient concentration (g kg^−1^), and the total nutrient uptake (kg ha^−1^) in the aboveground biomass was calculated from the summed nutrient uptake by each organ.

#### 2.3.5 Protein composition and processing quality

In 2020–2021 and 2021–2022, protein fractions from whole meal flour were extracted using a sequential extraction procedure based on Luo et al. (2019) with some modifications. Whole meal flour (0.50 g) was weighted to extract the albumin with 5 mL of pure water in a plastic centrifuge tube using the oscillation (20 min) and centrifugation (4,000 rpm for 7 min) method and repeated four times. The extract was collected as albumin after four cycles of oscillation and centrifugation. Similarly, the residue in the tube was extracted with another solution to obtain the fraction of globulin, gliadin, and glutenin using 2% NaCl, 75% ethanol, and 0.2% NaOH solution, respectively, using the same procedure as described above and repeated four times. Afterward, the concentration of protein fraction was determined by the Kjeldahl method (H8750, Haineng Company, China).

In 2020–2021 and 2021–2022, the processing quality including development time (min), stable time (min), sedimentation (mL), wet gluten (%), and extensibility (mm) were determined using a near-infrared analyzer (DA7250, Perten, Stockholm, Sweden).

#### 2.3.6 Resource use efficiency

Rainfall fallow efficiency (RFE, %) was calculated using the following equations (He et al., 2016c):


RFE=WH÷P

where WH (mm) is the water harvesting during the summer fallow season and P (mm) is the precipitation during the summer fallow season.

WUE (kg ha^−1^ mm^−1^) was calculated according to Zhao et al. (2023b):


WUE=Y÷ET

where Y (kg ha^−1^) is the grain yield and ET (mm) is the evapotranspiration over the whole winter wheat growing season. Because the sampling depth was not uniform between different years, the soil water storage 180- to 200-cm soil layer at maturity is calculated as 2/3 of the 180- to 210-cm soil layer in 2020–2021 and 2021–2022.

The nutrient uptake efficiency and nutrient internal efficiency were calculated using the following equations (Huang et al., 2017):


$$\text{Nutrient uptake efficiency}\;(\text{kg k}{\text{g}}^{–1})={\text{U}}_{\text{t}}÷\;{\text{F}}_{\text{P}}$$

$$\text{Nutrient internal efficiency}\;(\text{kgk}{\text{g}}^{–1})={\text{Y}}_{\text{g}}÷{\text{ U}}_{\text{t}}$$

where U_t_ is the total nutrient uptake of N, P, and K in the aboveground biomass (kg ha^–1^); F_P_ is the pure fertilizer rate for N, P, and K (kg ha^–1^); and Y_g_ is the wheat grain yield (kg ha^–1^).

### 2.4 Statistical analysis

Means of the data for each treatment were calculated by averaging the values for each plot. Differences among the means were determined by analysis of variance (ANOVA) and the least significant difference (LSD) test at *p* = 0.05 using the SPSS statistical software package (version 18, IBM Corp., Chicago, IL, USA). The graphs were prepared using Microsoft Excel 2010.

## 3 Results

### 3.1 Water harvesting

**Table 2** shows tillage practices that significantly affected water harvesting during the fallow season and fallow efficiency except for 2019–2020, while significantly affecting soil water storage at sowing (SWS_s_) in all 4 years. Compared to PTST, STSM increased water harvesting during the fallow season (WH) and rainfall fallow efficiency (RFE) in 2018–2019 and 2020–2021 with an increase of 5.0% and 7.0% over the 4 years; in turn, SRFSM significantly increased WH and RFE except for 2019–2020 with an increase of 9.6% and 12.2% over the 4 years. Thus, STSM and SRFSM significantly increased the SWS_S_ in all 4 years with an average increase of 3.3% and 5.8%, respectively. These results indicated that SRFSM increases water harvesting during the fallow season and thereby increasing the soil water storage at sowing in the F–W system.

**Table 2:** Water harvesting during summer fallow season and soil water storage at sowing under different tillage practices in 2018–2022.

Year	Tillage practice	Water harvesting during fallow season (mm)	Rainfall fallow efficiency (%)	SWS at sowing (mm)
2018–2019	PTST	143.5c	49.4c	522.9c
	STSM	156.5b	53.9b	535.9b
	SRFSM	166.5a	57.3a	545.9a
2019–2020	PTST	206.2a	46.0a	564.5c
	STSM	206.6a	46.1a	571.8b
	SRFSM	208.4a	46.5a	579.8a
2020–2021	PTST	129.4c	42.5c	524.0c
	STSM	153.0b	50.2b	559.2b
	SRFSM	163.5a	53.7a	576.0a
2021–2022	PTST	234.3b	32.8b	638.3c
	STSM	233.0b	32.6b	658.1b
	SRFSM	243.9a	34.1a	677.8a
4-year average	PTST	178.4c	42.7c	562.5c
	STSM	187.3b	45.7b	581.3b
	SRFSM	195.6a	47.9a	594.9a
*F* value	Year (Y)	2,955.4**	1,830.3**	6,081.2**
	Tillage practice (T)	163.5**	221.7**	722.5**
	Y×T	32.7**	58.2**	50.9**

PTST, plowing tillage with straw turnover; STSM, subsoiling with straw mulching; SRFSM, STSM coupled with RF. Different small letters after the data within the same column and each year indicate significant difference among treatments at p < 0.05. ** indicates statistical significance of variance at p < 0.01.

### 3.2 Yield components, yield, and harvest index

Both the year and tillage practices had a significant impact on the yield, yield components, and harvest index (HI) of wheat, as shown in **Table 3**. In comparison to PTST, STSM led to a notable increase in grain yield by 14.3%, with respective increases of 21.5%, 22.4%, and 11.2% in 2018–2019, 2020–2021, and 2021–2022. Similarly, SRFSM resulted in a 25.9% increase in grain yield, with increases of 26.8%, 9.7%, 29.9%, and 37.2% in 2018–2019, 2019–2020, 2020–2021, and 2021–2022. When averaged across the 4 years, both STSM and SRFSM significantly boosted spike numbers, 1,000-grain weight, grain yield, and biomass yield compared to PTST. Additionally, SRFSM showed a significant increase in grains per spike and HI. In comparison to STSM, SRFSM demonstrated significant improvements across all yield traits and achieved a 10.2% increase in grain yield.

**Table 3:** The yield components, yield, and harvest index of wheat under different tillage practices in 2018–2022.

Year	Tillage practice	Spike numbers (kg·hm^−2^)	Grains per spike	1,000-grain weight (g)	Grain yield (kg·hm^−2^)	Biomass yield (kg·hm^−2^)	Harvest index (%)
2018–2019	PTST	373.8c	31.6b	44.3b	5219c	11894b	49.4c
	STSM	453.6b	31.8b	44.6b	6342b	13915a	51.3b
	SRFSM	469.2a	34.8a	45.9a	6620a	14204a	52.4a
2019–2020	PTST	353.8b	30.5a	44.5c	5052b	12156b	46.8a
	STSM	350.2b	28.9b	47.1a	5049b	12387b	45.9ab
	SRFSM	363.7a	30.1a	45.9b	5544a	13726a	45.4b
2020–2021	PTST	381.7c	37.3b	48.2b	5944c	13562b	49.3a
	STSM	458.0a	36.6c	48.7ab	7277b	17596a	46.5b
	SRFSM	446.3b	38.6a	49.6a	7720a	17871a	48.6a
2021–2022	PTST	365.8c	27.4b	43.3b	4723c	11350c	46.8b
	STSM	420.8b	29.1a	44.7b	5251b	12874b	45.9b
	SRFSM	461.7a	29.6a	45.9a	6481a	15022a	48.5a
4-year average	PTST	368.8c	31.7b	45.1c	5234c	12241c	48.1b
	STSM	420.7b	31.6b	46.3b	5980b	14193b	47.4c
	SRFSM	435.2a	33.3a	46.8a	6591a	15206a	48.8a
*F* value	Year (Y)	379.7**	859.0**	95.1**	964.0**	608.3**	97.3**
	Tillage practice (T)	488.3**	64.6**	27.5**	972.5**	669.4**	12.9**
	Y×T	59.7**	17.2**	4.1**	79.5**	59.9**	11.4**

PTST, plowing tillage with straw turnover; STSM, subsoiling with straw mulching; SRFSM, STSM coupled with RF. Different small letters after the data within the same column and each year indicate significant difference among treatments at p < 0.05. ** indicates statistical significance of variance at p < 0.01.

### 3.3 Evapotranspiration and water use efficiency

As shown in **Table 4**, compared to PTST, STSM did not affect the 4-year average ET with a 2-year increase and a 1-year decrease, but significantly increased the WUE in 3 of 4 years with an average increase of 14.2%; SRFSM significantly increased the ET in all 4 years with an average increase of 3.0%, as well as WUE in all 4 years with an average increase of 22.6%. Compared to STSM, SRFSM significantly increased ET in 2 of 4 years and WUE in all 4 years, respectively, by 2.9% and 6.8% over the 4 years.

**Table 4:** The evapotranspiration and water use efficiency of wheat under different tillage practices in 2018–2022.

Tillage practice	Evapotranspiration (mm)					Water use efficiency (kg ha^−1^ mm^−1^)				
	2018–2019	2019–2020	2020–2021	2021–2022	4-year average	2018–2019	2019–2020	2020–2021	2021–2022	4-year average
PTST	271.6b	374.9a	396.1c	332.4b	343.7b	19.2c	13.5b	15.0c	14.2c	15.5c
STSM	277.7a	370.6a	410.0b	313.6c	343.0b	22.8b	13.6b	17.7b	16.7b	17.7b
SRFSM	281.4a	372.4a	418.1a	342.1a	353.5a	23.5a	14.9a	18.5a	18.9a	19.0a
*F* value										
Year (Y)	2,835.9**					982.1**				
Tillage practice (T)	40.6**					379.8**				
Y×T	22.1**					24.9**				

PTST, plowing tillage with straw turnover; STSM, subsoiling with straw mulching; SRFSM, STSM coupled with RF. Different small letters after the data within the same column and each year indicate significant difference among treatments at p < 0.05. ** indicates statistical significance of variance at p < 0.01, respectively.

### 3.4 Nutrient use efficiency

**Table 5** shows that STSM and SRFSM can increase N, P, and K uptake efficiency and P and K internal efficiency. Compared to PTST, the N, P, and K uptake efficiency were respectively increased by 16.9%, 16.2%, and 15.3% under STSM, as well as 32.5%, 29.7%, and 26.3% under SRFSM over the 2 years, while the P and K internal efficiency were also increased by 3.9% and 6.0% under SRFSM. In comparison to STSM, SRFSM significantly increased N, P, and K uptake efficiency, and K internal efficiency by 11.8%, 10.4%, 8.8%, and 4.7% over the 2 years.

**Table 5:** The N, P, and K uptake and internal efficiency of wheat under different tillage practices in 2020–2022.

Year	Tillage practice	Nutrient uptake efficiency (kg·kg^−1^)			Nutrient internal efficiency (kg·kg^−1^)		
		N	P	K	N	P	K
2020–2021	PTST	0.85c	0.45c	1.93c	37.4a	331.1b	61.8b
	STSM	1.03b	0.53b	2.33b	37.5a	344.8a	62.7ab
	SRFSM	1.09a	0.56a	2.45a	37.7a	345.7a	63.2a
2021–2022	PTST	0.70c	0.30c	1.87c	36.7a	395.3a	51.1b
	STSM	0.77b	0.34b	2.05b	36.3a	393.9a	51.4b
	SRFSM	0.94a	0.41a	2.35a	37.3a	408.8a	56.4a
2-year average	PTST	0.77c	0.37c	1.90c	37.0a	363.2b	56.4b
	STSM	0.90b	0.43b	2.19b	36.9a	369.4a	57.0b
	SRFSM	1.02a	0.48a	2.40a	37.5a	377.3a	59.8a
*F* value	Year (Y)	9,524.8**	13,883.7**	1,892.3**	107.6**	810.4**	1,660.6**
	Tillage practice (T)	5,334.5**	1,985.5**	7,328.1**	0.5	10.4*	33.1**
	Y×T	385.5**	98.2**	392.3**	1.3	2.1	12.5**

PTST, plowing tillage with straw turnover; STSM, subsoiling with straw mulching; SRFSM, STSM coupled with RF. Different small letters after the data within the same column and each year indicate significant difference among treatments at p < 0.05. * and ** indicate statistical significance of variance at p < 0.05 and p < 0.01, respectively.

### 3.5 Contents of protein components and processing quality

Both the year and tillage practices had a significant impact on the contents of protein and protein components in grain of wheat, as shown in **Table 6**. Averaged across the 2 years, compared to PTST, the contents of albumin, globulin, gliadin, glutenin, and total protein under STSM were increased by 8.6%, 1.7%, 10.4%, 7.6%, and 6.9%, respectively, as well as by 16.0%, 17.8%, 18.0%, 15.2%, and 15.2% under SRFSM. In comparison to STSM, SRFSM significantly increased those by 6.5%, 13.7%, 6.4%, 6.6%, and 7.2%, respectively. These results indicated that STSM and SRFSM during the fallow season increase the contents of grain protein and its components in dryland wheat.

**Table 6:** The content of protein and its components in grain of wheat under different tillage practices in 2020–2022.

Year	Tillage practice	Albumin/%	Globulin/%	Gliadin/%	Glutenin/%	Total protein/%
2020–2021	PTST	2.08c	1.87b	2.43c	3.55c	10.93c
	STSM	2.24b	1.87b	2.80b	3.93b	11.85b
	SRFSM	2.44a	2.24a	2.99a	4.30a	12.98a
2021–2022	PTST	1.67c	1.73b	3.14c	3.30c	10.83c
	STSM	1.82b	1.79b	3.34b	3.46b	11.41b
	SRFSM	1.90a	2.00a	3.57a	3.60a	12.07a
2-year average	PTST	1.87c	1.80b	2.78c	3.43c	10.88c
	STSM	2.03b	1.83b	3.07b	3.69b	11.63b
	SRFSM	2.17a	2.12a	3.28a	3.95a	12.53a
*F* value	Year (Y)	5,177.4**	3,055.2**	2,044.9**	27,712.9**	1,659.5**
	Tillage practice (T)	767.0**	5,190.5**	459.0**	11,258.7**	6,482.2**
	Y×T	38.6**	293.8**	13.7**	2,068.8**	387.5**

PTST, plowing tillage with straw turnover; STSM, subsoiling with straw mulching; SRFSM, STSM coupled with RF. Different small letters after the data within the same column and each year indicate significant difference among treatments at p < 0.05. ** indicates statistical significance of variance at p < 0.01.

Although they varied yearly, tillage practices significantly affected the processing quality of wheat (**Table 7**). Considering the 2-year average, with the exception of development time, STSM significantly increased all the measured processing quality of development time, stable time, sedimentation, wet gluten, and extensibility compared with PTST. Likewise, SRFSM significantly increased the processing quality compared with STSM.

**Table 7:** The grain processing quality of wheat under different tillage practices in 2020–2022.

Year	Tillage practice	Development time/min	Stable time/min	Sedimentation/mL	Wet gluten/%	Extensibility/mm
2020–2021	PTST	2.43a	1.40c	25.79c	27.57b	144.5c
	STSM	2.43a	2.10b	27.33b	28.42a	148.9b
	SRFSM	2.57a	2.33a	28.44a	28.74a	151.9a
2021–2022	PTST	3.76b	2.11c	17.95c	24.34c	129.1c
	STSM	3.85b	3.42b	19.41b	25.47b	131.3b
	SRFSM	4.08a	4.32a	20.40a	26.42a	136.4a
2-year average	PTST	3.10b	1.76c	21.87c	25.96c	136.8c
	STSM	3.14b	2.76b	23.37b	26.95b	140.1b
	SRFSM	3.32a	3.33a	24.42a	27.58a	144.1a
*F* value	Year (Y)	1,523.5**	498.9**	2,347.6**	752.1**	2,270.9**
	Tillage practice (T)	14.4**	235.0**	81.8**	83.6**	155.9**
	Y×T	2.2**	37.7**	0.1**	6.9**	4.4**

PTST, plowing tillage with straw turnover; STSM, subsoiling with straw mulching; SRFSM, STSM coupled with RF. Different small letters after the data within the same column and each year indicate significant difference among treatments at p < 0.05. ** indicates statistical significance of variance at p < 0.01.

### 3.6 Nitrate-N residue

**Figure 3:**
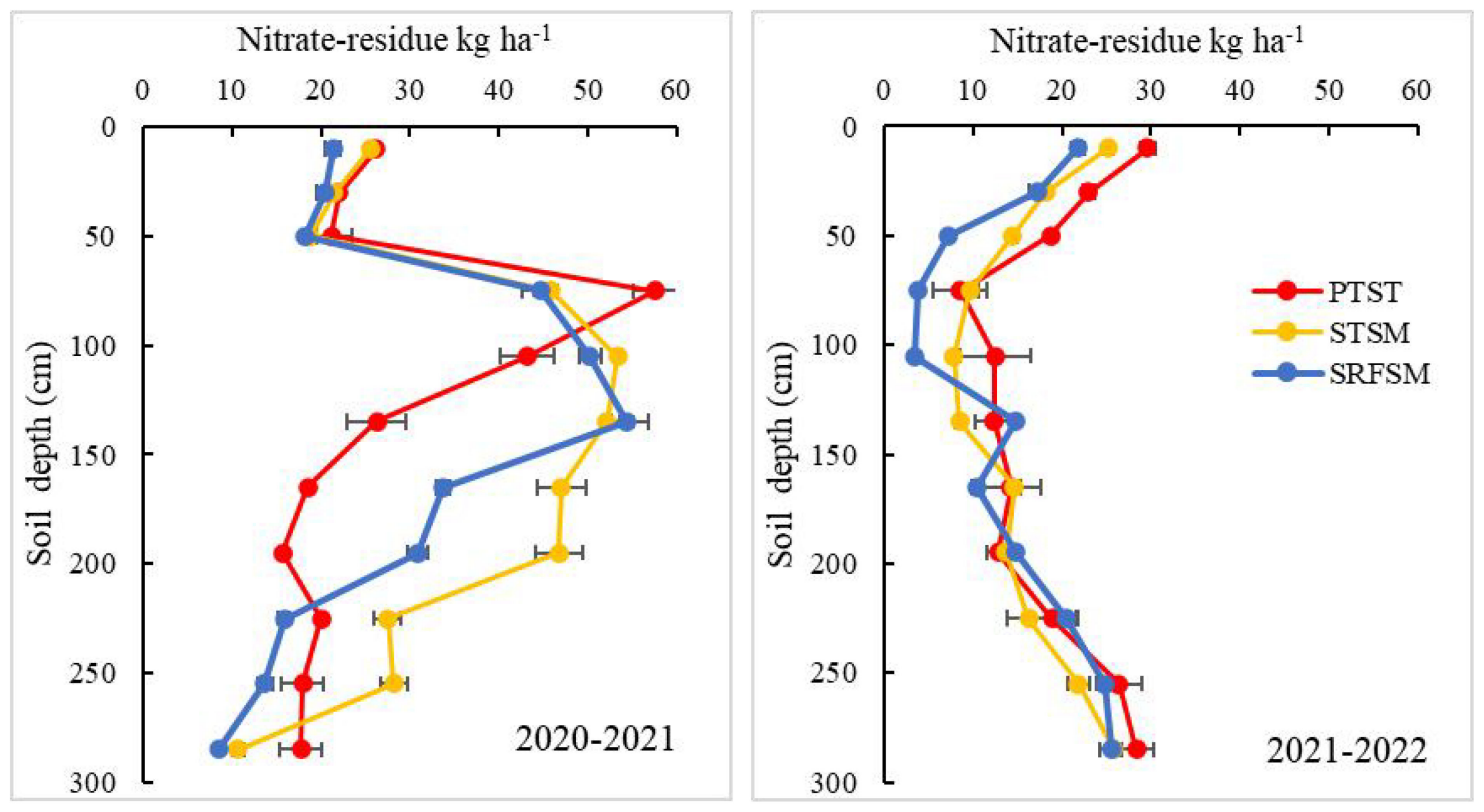
Effects of different tillage practices in summer fallow season on soil nitrate residue of wheat field in dryland. PTST, plowing tillage with straw turnover; STSM, subsoiling with straw mulching; SRFSM, STSM coupled with RF.

The nitrate-N residue level varied across soil depth, tillage practices, and years (**Figure 3**, **Table 8**). In comparison to PTST, both STSM and SRFSM significantly decreased the nitrate-N residue in the 0- to 60-cm soil layer in both years. However, they increased the nitrate-N residue in the 0- to 300-cm and the 60- to 180-cm soil layer in 2020–2021 but decreased in 2021–2022. Compared to PTST, STSM increased the nitrate-N residue by 12.5%, 23.8%, and 20.8% in the 0- to 300-cm, 60- to 180-cm, and 180- to 300-cm soil layer over the 2 years; however, SRFSM did not affect the nitrate-N residue in the 0- to 300-cm soil layer with a decrease of 24.6% in the 0- to 60-cm soil layer and increase of 11.7% in the 60- to 180-cm soil layer. In comparison to STSM, SRFSM significantly reduced the nitrate-N residue in the 0- to 300-cm soil layer by 16.1%, with a decrease of 16.4%, 10.9%, and 23.3% in the 0- to 60-cm, 60- to 180-cm, and 180- to 300-cm soil layer.

**Table 8:** The nitrate-N residues in the 0- to 300-cm soil layer under different tillage practices in 2020–2022.

Year	Tillage practice	0–300 cm	0–60 cm	60–180 cm	180–300 cm
2020–2021	PTST	286.4c	69.5a	145.4c	71.5b
	STSM	377.4a	65.8b	198.5a	113.2a
	SRFSM	312.1b	60.1c	183.0b	69.0b
2021–2022	PTST	205.5a	71.4a	47.7a	86.4a
	STSM	176.0b	57.8b	40.6ab	77.6b
	SRFSM	164.6b	46.2c	32.7b	85.7ab
2-year average	PTST	245.9b	70.4a	96.5c	79.0b
	STSM	276.7a	61.8b	119.5a	95.4a
	SRFSM	238.3b	53.1c	107.8b	77.4b
*F* value	Year (Y)	887.4**	126.1**	2,678.6**	0.0
	Tillage practice (T)	23.8**	283.4**	25.8**	0.5
	Y×T	52.5**	60.4**	52.4**	65.5**

PTST, plowing tillage with straw turnover; STSM, subsoiling with straw mulching; SRFSM, STSM coupled with RF. Different small letters after the data within the same column and each year indicate significant difference among treatments at p < 0.05. ** indicates statistical significance of variance at p < 0.01.

## 4 Discussion

### 4.1 Soil water, wheat yield, and efficiency affected by ST and RF techniques during the summer fallow season

In dryland regions of China, water shortage is one of the most important factors threatening wheat production, and optimizing the techniques for enhancing soil water and WUE plays an important role in ensuring food security (Gan et al., 2013; Wang et al., 2019). Previous studies have reported that the ST, SM, and RF techniques could increase soil water (Adil et al., 2022), which resulted in an excellent moisture condition for plant growth, tiller differentiation, and dry matter and nutrient accumulation and metabolism, and ultimately achieve the aim of high yield and high efficiency. The studies in drylands of southern Shanxi found that ST during the summer fallow season significantly increased fallow efficiency and soil water storage at sowing, and this could be maintained until the flowering stage, resulting in yield gain and a significant increase in WUE (Sun et al., 2014; Xue et al., 2017; Zhang et al., 2018). Another long-term experiment of He et al. (2016a); He et al. (2016b); He et al. (2016c) showed that RF soil surface with residual plastic film (formed by previous wheat cropping) during the summer season significantly increased rainfall harvesting, thus improving grain yield, WUE, N internal efficiency, and shoot P and K uptake. In our experiment, 4 years of continuous treatment revealed significant variations in grain yield, WUE, and N, P, and K uptake efficiency across different years. Notably, the highest values for these parameters were consistently observed under SRFSM, with STSM following closely behind. This trend suggests that the ST and RF techniques, showing sustainability and stability, are performing well at present. These improvements and sustainability were mainly ascribed to the optimized water harvesting during the fallow season attributed to the STSM and RF techniques, in accordance with the previous studies (Sun et al., 2014; He et al., 2016a, He et al., 2016b, He et al., 2016c; Xue et al., 2017; Zhang et al., 2018). In addition, the improvement in soil properties by subsoiling and/or straw mulching may explain these increases in grain yield and efficiency (Wang et al., 2015; Zhang et al., 2016). It is important to note that STSM and SRFSM did not significantly increase water harvesting (WH) and rainfall fallow efficiency (RFE), resulting in the lowest yield increase during the normal year of 2019–2020. These findings once again underscore the importance of rainfall harvesting during the summer fallow season for dryland wheat production. Above all, the employment of the combined agronomic techniques of STSM and RF during the summer fallow season could increase rainfall harvesting and dryland wheat productivity such as yield, water, and nutrient efficiency.

### 4.2 Grain quality affected by ST and RF during the summer fallow season

With the increased standards of living and the development of the food industry, the demand for high-quality grain and flour of wheat has increased rapidly (Lin et al., 2016; Luo et al., 2018; Hu et al., 2021). Proteins and protein components, especially glutenin and gliadin, which serve as multifunctional ingredients in processing and nutritional quality, are the key criteria for assessing the wheat quality in grain (Luo et al., 2019). Previous literature has shown that the effect of subsoiling on wheat quality is inconclusive. A study by Sun et al. (2014) reported that soil water had a positive correlation with grain globulin, gliadin, protein content, and yield. ST during the summer fallow period significantly enhanced soil water, thus increasing grain protein and its components, as well as protein yield in a humid year. A study on the tidal soil areas in China shows that ST significantly increased the protein content of wheat grain by 2.99%–4.90% (Jia et al., 2022). However, another study by Sun et al. (2013) indicated that ST during the summer fallow season decreased the content of albumin, gliadin, and total protein in grains, while increasing the globulin content, glutelin content, and protein yield. Additionally, Xue et al. (2017) reported that ST decreased the grain protein content compared to PT during the summer fallow season. However, a study in Romania by Cociu and Alionte (2017) concluded that ST decreased the protein content of wheat but increased that of maize. In our case, compared to PTST, the contents of protein, albumin, globulin, gliadin, and glutenin in wheat grain under STSM were significantly increased, and the effectiveness was uniform in the two experimental years (**Table 6**). This result indicated that subsoiling with straw mulching (STSM) during the summer fallow season can increase the content of protein and its components of wheat grain in dryland. The significantly increased N uptake efficiency (**Table 4**) may explain these results. A field study by Zhang et al. (2017) also found that ST during the summer fallow season significantly increased wheat grain protein by 7%, mainly due to the N uptake improvement caused by the well-water condition induced by ST. Luo et al. (2020) found that, on the basis of soil water management with a target relative content of 100% at wintering and jointing, RF significantly increased the protein and wet gluten content of wheat grain. However, He et al. (2016b) found that the RF soil surface with residual plastic film—formed by previous wheat planting—during the summer fallow season decreased the wheat grain N concentration by 8%, which was associated with the increased N requirement for grain N accumulation and decreased its internal efficiency. In our study, the application of the RF technique within SRFSM during the summer fallow season significantly increased the protein content and protein components of wheat grain. This result was consistent with Luo et al. (2020) but not consistent with He et al. (2016b). Variations in experimental conditions may account for differences in study outcomes.

Commercial value is also determined by the processing quality of wheat. With the gradual increase in bakery flour products, the dough rheological properties, as an important evaluation index of in bakery flour products and wheat processing quality, are being paid more and more attention in nowdays with the improvement of people's living standard. On the Loess Plateau, ST significantly increased sedimentation values and the gluten content of wheat grain (Sun et al., 2013), especially when combined with plastic film mulching (Zhao et al., 2017). Campiglia et al. (2015) reported that ST did not affect the gluten content of durum wheat in the Mediterranean environment of central Italy. However, to the best of our knowledge, few studies focus on how RF affects the processing quality of wheat. In this study, dough rheological properties such as development time, stable time, sedimentation, wet gluten, and extensibility showed the following order: SRFSM>STSM>PTST, with the significant differences between every two treatments, and the impact of different practices on wheat processing quality was the same in the two experimental years (**Table 7**). The improvement in dough rheological properties is mainly due to the increased protein content and its components (**Table 6**), as reported by Zhang et al. (2018). These results show that the processing quality of dryland wheat can be improved when STSM and SRFSM are applied during the summer fallow season. Accordingly, the results of this study provide a basis for further investigation and regulation on the quality of wheat in drylands, but further studies are needed to verify our findings in field experiments under varied soil and climatic conditions.

### 4.3 Soil nitrate-N affected by ST and RF during the summer fallow season

The nitrate-N present in the soil may contribute greatly to the N requirements of crops (Dai et al., 2015), but it is also susceptible to leaching, denitrification, and emission if the level exceeds the safe threshold (López-Bellido et al., 2013; Wang and Li, 2019). In this study, compared to PTST, STSM significantly decreased the nitrate-N residue in the 0- to 60-cm soil layer in both years, but increased it in the 60- to 300-cm soil layer. This is mainly ascribed to the increased N uptake efficiency. Previous studies showed that ST reduced the nitrate-N content in the 0- to 80-cm layer at maturity of winter wheat but increased it in the 120- to 160-cm soil layer in the North China Plain (Zheng et al., 2012; Wang et al., 2015). However, some studies showed that ST helps to increase the nitrate residue in the 0- to 80-cm soil layer but reduced it in the 0- to 380-cm soil layer (Wu et al., 2023). The present study also found that, compared to STSM, SRFSM significantly reduced the nitrate-N residue in the 0- to 300-cm soil layer by 16.1%, with a decrease of 16.4%, 10.9%, and 23.3% in the 0- to 60-cm, 60- to 180-cm, and 180- to 300-cm soil layers. This was mainly because RF increased soil water and promoted N uptake by wheat plants, and finally reduced nitrate-N residues in the wheat production system (Zhao et al., 2023a). However, another study in the summer maize–winter wheat double cropping system showed that RF planting patterns increased the nitrate-N residue in the 0- to 100-cm layer, but there was no difference in the 0- to 380-cm soil depth (Wu et al., 2023). Above all, the influence of ST and RF during the summer fallow season on nitrate-N residue remains uniform; thus, further studies are needed to verify the influence of ST coupled with RF on nitrate-N residue under varied environmental and cropping conditions.

Our results also showed that nitrate-N residues in soil were closely related to precipitation (**Figure 3**). In 2020–2021, the nitrate-N residue was 145.4–198.5 kg N ha^−1^ in the 60- to 180-cm soil depth, which surpassed 50% of that in the 0- to 300-cm soil profile. This suggests that the 60- to 180-cm soil layer was the nitrate-N enrichment region. However, the distribution of nitrate-N residue (especially in the 90- to 180-cm soil layer) in 2021–2022 was obviously different from 2020–2021 because of the high amount of precipitation from July to September 2021, where the nitrate-N leached deeper into the soil profile by 90 cm due to heavy rainfall. Most soil N is soluble and easily moves away from upper soil under the conditions of heavy rainfall during the summer season, resulting in the nitrate-N migrating to the even deeper 100- to 200-cm soil layer along the gaps formed by crop roots. In our case, the greater nitrate-N change between 2019–2020 and 2020–2021 was found under STSM and SRFSM compared to PTST. This result demonstrated that STSM and SRFSM have a higher risk of nitrate-N leaching under heavy rainfall conditions due to the increased ability of water infiltration. Zhao et al. (2023b) also reported that heavy rainfall resulted in more nitrate-N leaching in the study area. Hence, the effects of precipitation on the distribution of nitrate-N in soil should be considered in wheat production to effectively utilize soil nitrate-N and reduce nitrate-N leaching to deep soil layers.

## 5 Conclusion

The obtained results showed that the STSM and RF techniques during the summer fallow season can significantly increase rainfall harvesting during the summer fallow season and soil water storage at sowing. Water improvement under STSM and SRFSM increased water and nutrient uptake efficiency, and ultimately improved grain yield, protein content and its components, and most indicators of processing quality in wheat. In comparison to STSM, rainfall harvesting, grain yield, efficiency, protein content, and processing quality under SRFSM were significantly increased but soil nitrate-N residue was significantly decreased. This study indicated that using the combination of subsoiling and ridge and furrow soil surface during the summer fallow season (SRFSM) is a promising strategy to increase yield, quality, and efficiency, and reduce the soil nitrate-N residue in the dryland summer fallow–winter wheat rotation system.

## Data availability statement

The original contributions presented in the study are included in the article/supplementary material. Further inquiries can be directed to the corresponding authors.

## Author contributions

JW: Conceptualization, Data curation, Investigation, Writing – original draft, Writing – review & editing. RW: Data curation, Investigation, Visualization, Writing – review & editing. WZ: Data curation, Investigation, Visualization, Writing – review & editing. KZ: Data curation, Investigation, Visualization, Writing – review & editing. SW: Data curation, Investigation, Visualization, Writing – review & editing. JZ: Data curation, Investigation, Visualization, Writing – review & editing. HW: Writing – review & editing. GF: Writing – review & editing. MH: Conceptualization, Funding acquisition, Writing – review & editing. YL: Conceptualization, Funding acquisition, Writing – review & editing.
